# Cervical Lymph Nodes Detected by F-18 FDG PET/CT in Oncology Patients: Added Value of Subsequent Ultrasonography for Determining Nodal Metastasis

**DOI:** 10.3390/medicina56010016

**Published:** 2019-12-31

**Authors:** Seokho Yoon, Kyeong Hwa Ryu, Hye Jin Baek, Tae Hoon Kim, Jin Il Moon, Bo Hwa Choi, Sung Eun Park, Ji Young Ha, Dae Hyun Song, Hyo Jung An, Young Jin Heo

**Affiliations:** 1 Department of Nuclear Medicine and Molecular Imaging, Gyeongsang National University School of Medicine and Gyeongsang National University Changwon Hospital, 11 Samjeongja-ro, Seongsan-gu, Changwon 51472, Korea; yoon.seokho@gmail.com; 2 Department of Radiology, Gyeongsang National University School of Medicine and Gyeongsang National University Changwon Hospital, 11 Samjeongja-ro, Seongsan-gu, Changwon 51472, Korea; ryukh0329@gmail.com (K.H.R.); drlotus@naver.com (J.I.M.); iawy82@gmail.com (B.H.C.); uneyes@hanmail.net (S.E.P.); wonpiece@gmail.com (J.Y.H.); 3 Department of Radiology, Institute of Health Sciences, Gyeongsang National University School of Medicine, 816-15 Jinju-daero, Jinju 52727, Korea; 4 Department of Internal Medicine, Gyeongsang National University School of Medicine and Gyeongsang National University Changwon Hospital, 11 Samjeongja-ro, Seongsan-gu, Changwon 51472, Korea; plm.dr.th.kim@gmail.com; 5 Department of Pathology, Gyeongsang National University School of Medicine and Gyeongsang National University Changwon Hospital, 11 Samjeongja-ro, Seongsan-gu, Changwon 51472, Korea; golgy@hanmail.net (D.H.S.); ariel2020@naver.com (H.J.A.); 6 Department of Radiology, Busan Paik Hospital, Inje University College of Medicine, 75, Bokji-ro, Busanjin-gu, Busan 47392, Korea

**Keywords:** lymph node, metastasis, neck, PET/CT, ultrasound

## Abstract

*Background and Objectives*: To investigate the diagnostic performance of F-18 fluorodeoxyglucose positron emission tomography/computed tomography (PET/CT) and subsequent ultrasonography (US) for determining cervical nodal metastasis in oncology patients. *Materials and Methods*: Fifty-nine cervical lymph nodes (LNs) initially detected by PET/CT with subsequent neck US were included in this retrospective study. All LNs were subjected to US-guided fine-needle aspiration or core needle biopsy. The maximum standardized uptake value (SUVmax) and sonographic features were assessed. *Results*: Forty-three of 59 cervical LNs detected by PET/CT were malignant. PET/CT alone showed a highest diagnostic value for metastatic LNs with 81.4% sensitivity, 68.8% specificity, and 78% accuracy when SUVmax ≥5.8 was applied as an optimal cut-off value. Combined PET/CT and subsequent US diagnoses for determining nodal metastasis showed the following diagnostic performance: 81.4% sensitivity, 87.5% specificity, and 83.1% accuracy. There was a significant difference in the diagnostic performance between the two diagnostic imaging approaches (*p* = 0.006). *Conclusions*: Combined diagnosis using subsequent US showed a significantly higher diagnostic performance for determining nodal metastasis in the neck. Therefore, we believe that our proposed diagnostic strategy using subsequent US can be helpful in evaluating cervical LNs on PET/CT. Moreover, our results clarify the need for US-guided tissue sampling in oncology patients.

## 1. Introduction

The accurate diagnosis of cervical lymph node (LN) metastasis is important in oncology patients. In patients with head and neck malignancies, cervical LN metastasis is one of the most significant prognostic factors that affect the therapeutic strategy. The presence of cervical LN metastasis is also important in the non-head and neck malignancies because it indicates distant metastasis, which alters the stage and prognosis [1].

In clinical practice, 18-F fluorodeoxyglucose (FDG) positron emission tomography/computed tomography (PET/CT) is widely used for initial tumor staging, evaluation of treatment response, and detection of tumor recurrence [2,3]. Consequently, the number of PET/CT detected suspicious LNs is increasing, particularly in the neck [2]. However, benign lymphadenopathy may also reveal hypermetabolism mimicking metastasis because PET/CT provides information about tissue glucose metabolism. Therefore, a cytopathological examination is often required to determine the nature of PET/CT detected LNs for accurate diagnosis through subsequent ultrasonography (US) and US-guided tissue sampling.

Nevertheless, performing US-guided tissue sampling of all LNs detected on PET/CT is invasive for patients and not cost-effective. If subsequent US is helpful to predict metastasis in PET/CT detected cervical LNs, unnecessary invasive diagnostic procedures can be reduced. Although there are several previous studies evaluating the diagnostic performance of PET/CT, CT, MRI, and US for detecting cervical LN metastasis [4,5,6,7,8,9,10,11], there are few studies that provide helpful guidelines for identifying patients who need US-guided tissue sampling for pathological confirmation. Therefore, it is not clearly established whether pathological confirmation is required in all positive cervical LNs on PET/CT or if US-guided tissue sampling should be performed selectively in suitable patients according to subsequent ultrasound findings.

To the best of our knowledge, there is only one study that established the diagnostic workflow using subsequent US for PET-detected LNs in patients with various primary malignancies, even though it was confined to supraclavicular LNs [4]. Therefore, the purpose of this study was to investigate the diagnostic performance of subsequent US for determining metastasis in PET/CT positive cervical LNs of oncology patients and to clarify the need for US-guided tissue sampling. In the present study, we focused on the subsequent diagnostic procedure for positive cervical LNs detected on PET/CT.

## 2. Materials and Methods

### 2.1. Study Populations

We retrospectively reviewed electronic medical records to find consecutive patients matched from July 2016 to May 2018 with the following criteria: (1) patients having suspicious metastatic cervical LNs detected on PET/CT; (2) patients who underwent subsequent neck US; and (3) patients who underwent US-guided fine needle aspiration (US-FNA) or US-guided core needle biopsy (US-CNB) of the suspicious LNs detected on PET/CT. Based on these criteria, we selected 68 patients, and subsequently excluded 12 patients with hematologic malignancies. The final 56 patients comprised of 35 men and 21 women with a mean age of 63 ± 12.1 years (range, 32–90 years). The average interval between PET/CT and subsequent US was 19.7 ± 25.7 days (range, 0–47 days). The most common primary site of malignancies was lung cancer (n = 20), followed by laryngeal, tongue, tonsil, and gastric cancer (n = 4 for each cancer). Patient demographics and clinicopathologic variables are shown in Table 1.

### 2.2. Imaging Techniques of PET/CT and US 

FDG PET was performed in the PET/CT unit (Discovery PET/CT 710, GE Healthcare, Milwaukee, WI, USA). All patients fasted for at least 6 hours before imaging, and the glucose levels in peripheral blood were confirmed to be 150 mg/dL or less before F-18 FDG injection. A dose of approximately 4.44 MBq/kg of body weight of F-18 FDG was administered IV 1 hour before image acquisition. After the initial low-dose CT study (15–200 mA on auto-mA mode, 120 kVp), a standard PET protocol was used to scan from the skull to the proximal thighs with an acquisition time of 2 minutes per bed position in the 3D mode. Images were then reconstructed using ordered subset expectation maximization algorithm (2 iterations, 24 subsets).

Subsequent US for evaluating PET/CT positive LNs were performed by two radiologists with the EPIQ 7 unit (Philips Medical System, Bothell, WA) using a linear high-frequency probe (5–12 MHz frequency range). After US examination, US-FNA or US-CNB were performed in all included LNs for confirmative diagnosis. US-FNA was performed using a 23-gauge needle attached to a 10-mL disposable plastic syringe combining capillary action and suction aspiration techniques depending on the specific characteristics of the lesions. The specimens were immediately fixed in 95% ethanol. US-CNB was performed using disposable 18-gauge double action spring-activated needles (1.1 or 1.6 cm excursion; TSK AceCut; Create Medic, Yokohama, Japan). The obtained specimens were sent to the pathology department for microscopic examination.

### 2.3. Image Analysis

PET/CT images were reviewed at interactive workstations by one nuclear medicine physician with 4 years of experience. The reader was blinded to the results of other imaging modalities and cytopathologic results at the time of review. For semiquantitative analysis, F-18 FDG uptake was quantified for cervical LNs with the highest 18F-FDG uptake using standardized uptake value (SUV). For measurement of SUVs, a spherical volume of interest encompassing the complete lesions was defined using the AW Volume Viewer Software (GE Healthcare). The highest SUV(SUVmax) within the VOI was recorded.

US images were retrospectively reviewed in consensus by two neuroradiologists with 8 and 3 years of experience, who were blinded to other imaging results or clinical data. We recorded the shortest diameter (Dmin) of the targeted LNs. We defined suspicious findings for malignant LNs as follows: increased size (short-axis diameter >0.8 cm), loss of fatty hilum, hypoechogenicity or heterogeneous internal echogenicity (with respect to the surrounding muscles or fat; and/or inconsistent LN texture, except hyperechoic hilum), and round shape (short/long axis ratio >0.5) [12,13,14,15]. We used the histopathologic results of US-FNA or US-CNB as the standard reference for assessing the diagnostic performance of each image modality. Combined interpretation of PET/CT with subsequent US was also performed to ascertain the diagnostic performance of identifying nodal metastasis in the included LNs. In combined interpretation, we considered a cervical LN to be metastatic when it showed suspicious finding on both PET/CT and subsequent US.

### 2.4. Statistical Analysis

The data were tested for normal distribution using a Kolmogorov–Smirnov test. Continuous variables were expressed as a mean ± standard deviations (SD). Normally distributed variables were analyzed using an independent t-test. Group comparisons of categorical variables were performed by the χ^2^ test and Fisher’s exact test. Receiver operating characteristic (ROC) curve analysis was applied to obtain the optimal cut-off value of SUVmax and the number of suspicious US findings for differentiating metastatic LNs from benign LNs. A cut-off value for each variable was determined by maximizing the sum of sensitivity and specificity. The χ^2^ test for trend was used to evaluate the linear association between the number of suspicious US features and the frequency of nodal metastasis.

In addition, an ROC curve analysis was performed to compare the diagnostic performance of PET/CT alone or combined with subsequent US in terms of sensitivity, specificity, positive predictive value (PPV), negative predictive value (NPV), and accuracy of determining metastatic LNs. The area under the ROC curve (AUC) was compared using the method described by DeLong et al. [16]. All statistical analyses were performed using SPSS version 24.0 (IBM Corporation, Armonk, NY, USA) and MedCalc version 14.10 (MedCalc Software, Ostend, Belgium); *p* < 0.05 was considered statistically significant.

In the present study, all retrospective data collection and analyses were performed in accordance with the local institutional review board (IRB) guidelines after obtaining its approval. The IRB determined that patient approval and informed consent were not required for retrospectively reviewing images and electrical medical records. The patient’s records and information were anonymized and de-identified prior to analysis. 

## 3. Results

### 3.1. Study Population

A total of 59 cervical LNs in 56 patients were included, located in level I (4, 6.8%), II (21, 35.6%), IV (11, 18.6%), V (1, 1.7%), and supraclavicular fossa (22, 37.3%). Of the 59 included LNs, US-FNA was performed in 45 LNs (76.3%) and US-CNB in 14 LNs (23.7%).

Of these, 43 LNs (72.9%) were confirmed to be malignant and 16 LNs (27.1%) were benign on histopathological examination. The mean age of patients with nodal metastasis was significantly higher than that of the benign group: 64.5 ± 11.3 years vs. 59.4 ± 15.1 years (*p* = 0.034). In addition, mean Dmin of the LNs showed a significant difference between the two groups according to pathological results: metastasis, 1.3 ± 0.7 cm vs. benign, 0.6 ± 0.2 cm (*p* < 0.001).

### 3.2. PET/CT Findings

**Figure 1:**
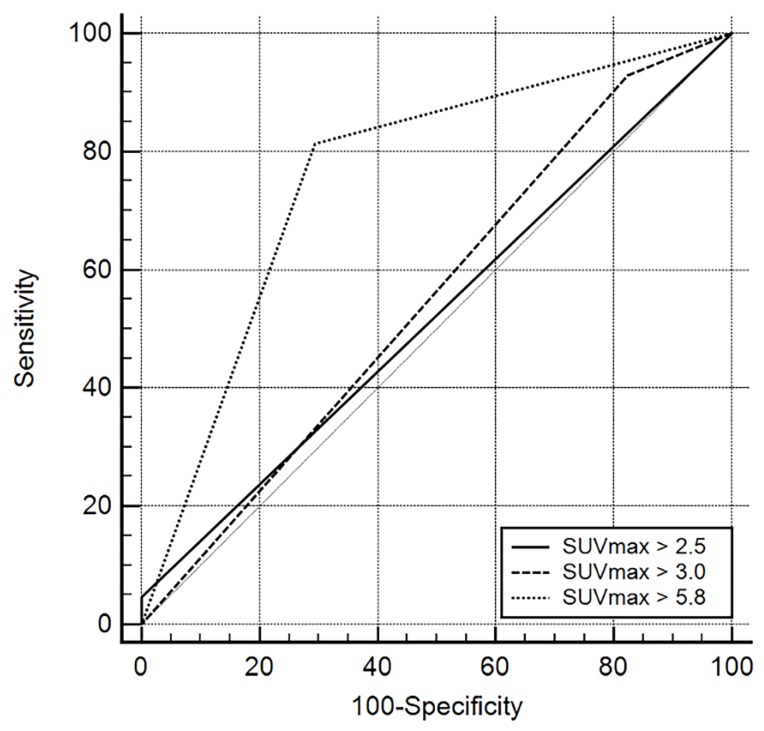
Comparisons of receiver operating characteristic (ROC) curves representing the diagnostic performance of PET/CT according to cut-off maximum standardized uptake values (SUVmax) (2.5, 3.0, and 5.8) for identifying metastatic lymph nodes. Diagonal line = 50% of the area under the ROC curve (AUC), and refers to a hypothetical marker that has no discriminatory power for differentiating metastasis from benign lymphadenopathy: SUVmax = 2.5 [AUC 0.523 (95% confidence interval {CI} 0.389 to 0.655)], SUVmax = 3.0 [AUC 0.559 (95% CI 0.424 to 0.688)], and SUVmax = 5.8 [AUC 0.751 (95% CI 0.621 to 0.854)].

The mean SUVmax value of the cervical LNs that were confirmed to be malignant was 11.33 ± 6.38 (range, 1.6–33.4), whereas that of benign lesions was 4.57 ± 1.64 (range, 2.7–7.4). SUVmax values of metastatic LNs were significantly higher than those of benign LNs (*p* < 0.001). With application of the best discriminative SUVmax cut-off value of 5.8, the diagnostic accuracy of PET/CT was 78% with sensitivity of 81.4%, specificity of 68.8%, PPV of 87.5%, and NPV of 57.9% (AUC, 0.751; 95% CI, 0.621 to 0.854, *p* = 0.002). We also compared ROC curve analyses between the cut-off values of SUVmax = 5.8 in the current study and SUVmax values, 2.5 and 3.0, suggested in other studies [4,5,6,7]. The diagnostic performance was significantly better when the cut-off value of SUVmax was 5.8 (Figure 1).

### 3.3. Subsequent US Findings

The US findings of cervical LNs are summarized in Table 2 and Table 3. All suspicious US findings were more frequently observed in metastatic LNs compared to benign ones (*p* <0.001). The χ^2^ test for trend also showed a linear-by-linear association between the number of suspicious US features and frequency of nodal metastasis (*p* <0.001). In the ROC analysis, we obtained an optimal cut-off number of suspicious US features for determining nodal metastasis, which was ≥2 with the highest diagnostic values as follows: accuracy 88.1%, sensitivity 97.7%, specificity 62.5%, PPV 87.5%, and NPV 90.9% (AUC, 0.801; 95% CI, 0.676 to 0.894, *p* <0.001). Therefore, we considered a cervical LN to be metastatic when it had two or more suspicious findings for malignancy on the US images.

### 3.4. Combined Diagnosis Using Subsequent US Findings

**Figure 2:**
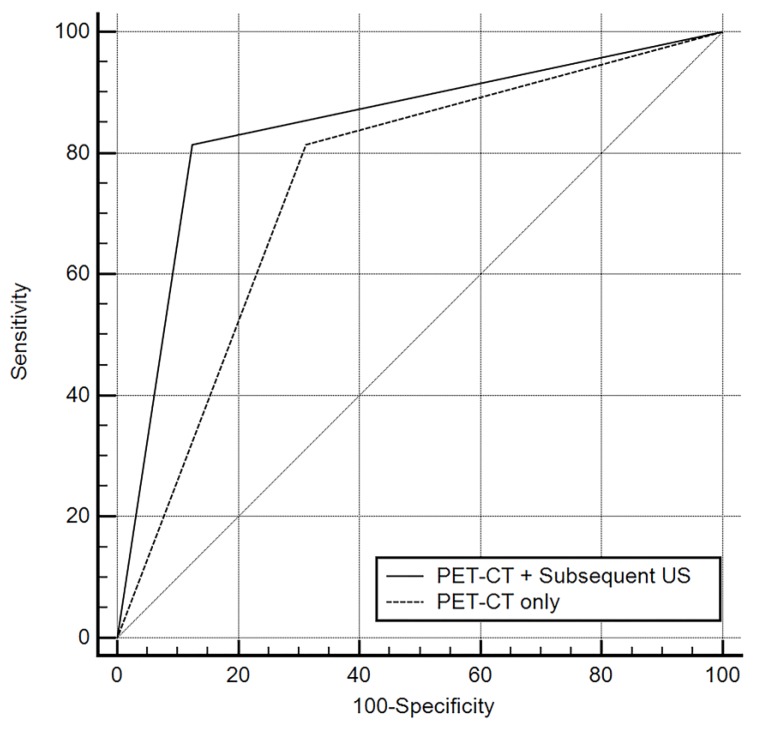
Comparisons of receiver operating characteristic (ROC) curves representing the diagnostic performance of each diagnostic modality for identifying metastatic lymph nodes. Diagonal line = 50% of the area under the ROC curve and refers to a hypothetical marker that has no discriminatory power for differentiating metastasis from benign lymphadenopathy.

The diagnostic performance of combined diagnosis using both PET/CT and subsequent US was: accuracy, 83.1%; sensitivity, 81.4%; specificity, 87.5%; PPV, 94.6%; and NPV, 63.6% (AUC, 0.844; 95% CI, 0.727 to 0.926). Comparing the diagnostic performance of the two approaches, combined diagnosis using subsequent US had a higher diagnostic value with statistically significant differences (*p* = 0.006; Table 4, Figure 2). The use of subsequent US improved the specificity, PPV, NPV and accuracy while preserving the sensitivity compared to diagnosis with PET/CT alone.

**Figure 3:**
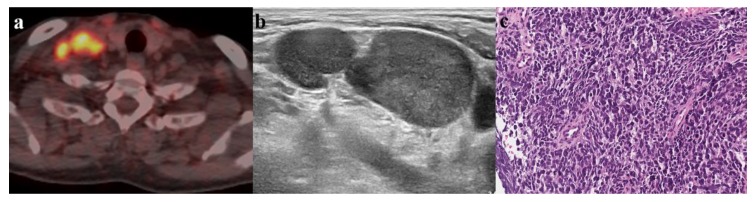
A 69-year-old man with lung cancer. (**a**) PET/CT shows focal areas of increased fluorodeoxyglucose uptake with a maximum standardized uptake value of 10 in the right supraclavicular area. (**b**) The corresponding lymph node (LN) demonstrates suspicious sonographic features as follows: heterogeneous cortical echogenicity, round shape, loss of fatty hilum and a short axis diameter of 1.7 cm. (**c**) Ultrasonography-guided core needle biopsy was performed for the suspicious LN. The LN is replaced by dense infiltration of tumor cells on the histopathologic examination (Hematoxylin and Eosin, ×200), and the tumor cells are composed of hyperchromatic and salt and pepper-like nuclei with scanty cytoplasm. Therefore, the LN was confirmed to be metastatic.

**Figure 4:**
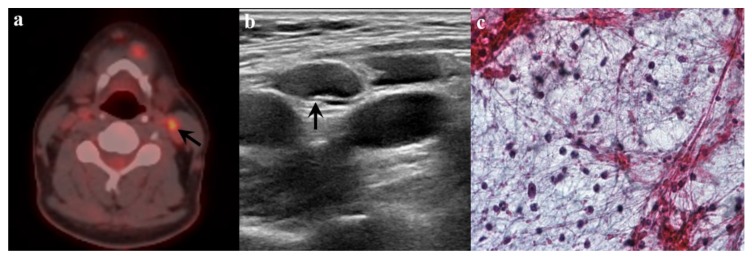
A 52-year-old man with tongue cancer. (**a**) PET/CT shows focal areas of increased fluorodeoxyglucose uptake with a maximum standardized uptake value of 5.9 in the left level II. (**b**) Ultrasonography revealed a benign-looking lymph node with normal cortical echogenicity, ovoid shape, preservation of fatty hilum and a short axis diameter of 0.6 cm. (**c**) Ultrasonography-guided fine needle aspiration was performed, and cytologic examination showed benign lymphoid cells scattered in the granular background. Epithelial cell clusters for suspicious malignancy were not found.

When the cut-off value of SUVmax was applied as 5.8, 40 of the 59 LNs (67.8%) were positive for malignancy (Figure 3). There were five false positive LNs that were classified as benign (n = 3) and metastatic (n = 2) on the subsequent US; however, these five LNs were finally confirmed as benign upon pathological examinations (Figure 4). These LNs had a mean Dmin of 0.7 cm (range, 0.6–0.9 cm) with a mean SUVmax of 6.8 (range, 6.1–7.4). On the other hand, all PET/CT negative LNs with less than SUVmax <5.8 (19/59, 32.2%) were assessed on subsequent US as follows: benign (8/19, 42.1%) vs. metastasis (11/19, 57.9%). The mean SUVmax of the nineteen LNs was 3.7 (range, 1.6–5.7) with a mean Dmin of 0.6 cm (range, 0.2–2.3 cm). Of the eight LNs with both PET/CT and US negative findings, only one LN had confirmed metastasis. In contrast, of the 11 LNs with PET/CT negative and US positive findings, four finally proved to be benign (36.4%) and seven to be metastatic (63.6%).

**Figure 5:**
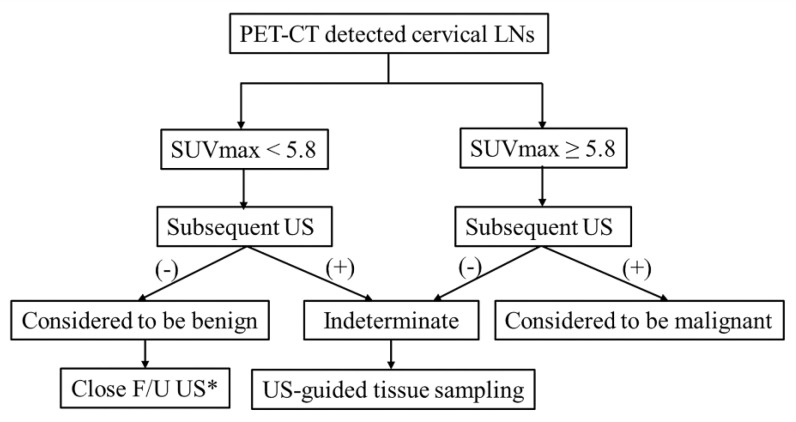
Schematic diagram suggesting diagnostic workflow of PET/CT-detected cervical lymph nodes. * US-guided tissue sampling can be selectively considered only for the LNs, which can change the tumor stage or method of operation. F/U, follow-up; LN, lymph node; SUVmax, maximum standardized uptake value; US, ultrasonography.

Based on the results of current study, we propose a diagnostic strategy in Figure 5. Because SUVmax can be influenced by the nodal size and types of primary tumor, it would be better to perform subsequent US to identify nodal metastases and the need for US-guided tissue sampling. If SUVmax >5.8 and subsequent US is positive, it would be reasonable to consider the LN as metastatic without the additional need for US-guided tissue sampling. If SUVmax < 5.8 and subsequent US is negative, close follow-up is recommended without the need for US-guided tissue sampling. US-guided tissue sampling is only recommended for the indeterminate category which means a discordant result between PET/CT and subsequent US. When this flow diagram strategy was applied to this study population, we found that the rate of misdiagnosis lowered to nearly one quarter with performing subsequent US compared to that with PET/CT alone: 3/59 (5.0%) vs. 13/59 (22%), respectively. In addition, 14 LNs with PET/CT and US discrepancy (14/59, 23.7%) were classified in the indeterminate category, of which seven were finally confirmed as benign (50%) and seven as metastatic (50%).

## 4. Discussion

The findings of our study indicate that the use of subsequent US in the PET/CT positive cervical LNs improved the diagnostic efficacy of determining nodal metastasis. We also defined the optimal cut-off values for SUVmax and the number of suspicious US features for determining nodal metastasis. Based on these results, we propose a novel diagnostic strategy to select appropriate candidates for US-guided tissue sampling among patients with PET/CT positive cervical LNs.

It is well known that PET/CT is a functional imaging technique that is highly sensitive in detecting primary and recurrent malignant tumors in the head and neck [17]. This is because FDG, a glucose analog, is an indicator of tumor viability based on the fact that malignant nodes have higher glucose utilization than normal nodes [18]. PET/CT is usually performed during the initial staging work-up of malignancy because of the high sensitivity of small malignant foci; therefore, the incidences of PET/CT detected cervical LNs are increasing. However, there are potential limitations of PET/CT for detecting metastatic LNs due to its limited resolution and partial volume effect, resulting in false negative or false positive results for detection of small LNs [19]. Furthermore, nodal necrosis is not likely to be detected by PET/CT because of its low glycolytic activity [20] and the benign inflammatory process may yield a false positive finding [21,22]. Because of these limitations, US-guided tissue sampling such as US-FNA or US-CNB is often required for the confirmative diagnosis of PET/CT detected LNs. Therefore, clinicians frequently rely on other imaging modalities such as US, CT and MRI to obtain ancillary information subsequent to PET/CT in daily clinical practice. In oncology patients, US has several advantages over other imaging modalities: (1) there is no risk of radiation exposure, even after multiple examinations; (2) the accessibility is higher than that of CT, MRI or PET/CT, and necessary scan time is shorter than that of MRI or PET/CT; and (3) the clinician can perform US-guided tissue sampling when a suspicious lesion is detected during the examination, which is the most important advantage.

In the current study, the diagnostic performance of combined diagnosis using subsequent US was significantly higher than that of PET/CT alone. The use of subsequent US for determining nodal metastasis improved most diagnostic indices, except for sensitivity, which was similar between the two approaches. These results support the notion that subsequent US is an effective diagnostic tool for determining nodal metastasis in the neck. Indeed, the use of subsequent US decreased the rate of misdiagnosis near to one quarter of that with PET/CT alone (5.0% vs. 22%). We also found that the optimal cut-off value of SUVmax for identifying cervical LN metastasis was 5.8, which was higher than that reported in other previous studies [4,5,6,7,11,23,24]. The reason behind this discrepancy is unclear; however, it may be related to the diverse type and composition of primary malignancies and the differences in PET/CT systems and protocols among the studies. However, the diagnostic performance showed highest values using SUVmax of 5.8, compared to previously reported cut-off values when applied on our study population.

In addition, we analyzed the optimal number of suspicious US findings for characterizing nodal metastasis in the PET/CT positive LNs. The presence of two or more suspicious US findings was the best predictor of nodal metastasis in PET/CT positive LNs in this study. In contrast to our results, a previous study considered PET/CT detected supraclavicular LNs to be malignant with any number of suspicious US findings without an optimal cut-off [4]. Therefore, we tried to objectively demonstrate the diagnostic ability of subsequent US in PET/CT detected cervical LNs.

In the present study, the mean SUVmax and Dmin of all 19 PET/CT negative LNs (including seven pathologically proven metastatic LNs) were lower than those of pathologically proven metastatic LNs. This is consistent with previous results showing that FDG uptake is often inconspicuously delineated in the small or necrotic lesions due to the partial volume effect and spatial resolution that can mask the actual metabolic activity of the lesion [19,20]. In addition, the five false-positive LNs on PET/CT also showed lower mean SUVmax and Dmin values than pathologically proven metastatic LNs. This finding is also similar to previous studies suggesting that a benign inflammatory reaction may produce false positive results on PET/CT [21,22,25]. Because F-18 FDG is not a tumor-specific tracer and acts as a basic energy substrate for many tissues, it can also accumulate in a variety of benign processes, including inflammatory conditions and reactive LNs [21,22,25].

A recent study argues that neck US has no additional value over negative PET/CT for diagnosing cervical LN metastases in esophageal cancer [26]. However, this study had a limitation on the generalizability of its results for the following reasons: (1) it was confined to esophageal cancer with various TNM staging; (2) it did not apply the clinically available cut-off value of SUVmax for determining metastasis; (3) the incidence of cervical LN metastasis in patients with esophageal cancer is not uncommon (up to 23%) owing to the dual embryologic origin of lymphatic pathways from branchiogenic and body mesenchyme, resulting in lymphatic interconnections among the three segmental drainage regions of the esophagus [27]; and (4) in general, only PET/CT positive LNs are of interest in oncology patients for the clinician to decide whether to perform additional examinations using other imaging modalities or tissue confirmation.

Therefore, in the present study, we propose a diagnostic workflow for PET/CT detected cervical LNs in various primary malignancies for selecting suitable patients who need US-guided tissue sampling. Herein, the presence of indeterminate category is inevitable in our diagnostic workflow because of discordant results between PET/CT and subsequent US in a clinical situation. In such cases, US-guided tissue sampling is needed to establish an accurate diagnosis and treatment planning. In addition, it is difficult to reassure in cases with a concordant negative result on both PET/CT and subsequent US of the cervical LNs; therefore, we suggest that patients in this category should be closely followed-up using imaging surveillance.

Several limitations of this study should be considered when interpreting the results. First, there was unavoidable selection bias because the data from all patients were evaluated retrospectively. Second, the sample size was small with a high incidence of nodal metastasis and various kinds of primary malignancies. Therefore, the results did not provide cancer-specific information for evaluating nodal metastasis in the neck. Further prospective studies are required to propose a cancer-specific diagnostic workflow using combined imaging modalities with reliable diagnostic performance. However, our results may be helpful to develop a generalized guideline for evaluating cervical LNs in various kinds of malignancies. Third, we only included cervical LNs detected on PET/CT in this study. This could also lead to a bias for evaluating FDG uptake and calculating SUVmax values. Fourth, there were few false-positive and false-negative results on PET/CT. Lastly, there was no case of surgical excisional biopsy, which may affect the results even though US-guided tissue sampling is a well-known first-line investigation of choice that is safe, cost effective, and fast with a high diagnostic efficacy in cervical LNs [28,29,30]. We expect that further studies with larger sample sizes and various targeted patients will validate our results in the near future.

## 5. Conclusions

This study reveals that the efficacy of combined diagnosis using subsequent US is greater than that of PET/CT alone for identifying cervical LN metastasis. Therefore, subsequent US for PET/CT detected cervical LNs can be helpful in determining the staging workup and management plan of oncology patients. We propose a diagnostic strategy for PET/CT detected cervical LNs and believe that this strategy can be helpful to properly classify oncology patients based on their need for US-guided tissue sampling, even though these diagnostic methods cannot completely replace tissue sampling for staging and treatment planning.

## Figures and Tables

**Table 1:** Patient Characteristics.

Character	Value
Sex	
Male	35 (62.5 %)
Female	21 (37.5 %)
Age	
Mean ± SD	63 ± 21.1
Range	32–90
Size (Dmin)	
Mean ± SD	1.1 ± 0.7
Primary tumor sites	
Lung cancer	20 (35.7 %)
Laryngeal cancer	4 (7.1 %)
Tongue cancer	4 (7.1 %)
Tonsil cancer	4 (7.1 %)
Gastric cancer	4 (7.1 %)
Salivary gland cancer	3 (5.4 %)
Hypopharyngeal cancer	3 (5.4 %)
Nasopharyngeal cancer	2 (3.6 %)
Thyroid cancer	2 (3.6 %)
Pancreas cancer	1 (1.8%)
Prostate cancer	1 (1.8%)
Skin cancer	1 (1.8%)
Ureter cancer	1 (1.8%)
Breast cancer	1 (1.8%)
Cholangiocarcinoma	1 (1.8%)
Colon cancer	1 (1.8%)
Esophageal cancer	1 (1.8%)
Myxoinflammatory fibroblastic sarcoma	1 (1.8%)
Malignancy of unknown origin	1 (1.8%)

Dmin, minimal diameter; SD, standard deviation.

**Table 2:** Diagnostic performance of ultrasonography in the assessment of lymph node metastasis.

Imaging Characteristics	% (No. with Positive Findings/Total No.)			
	Malignant	Benign	Total	*p* Value
Increased size (short-axis diameter >0.8 cm)	65.1 (28/43)	18.8 (3/16)	52.5 (31/59)	0.005
Loss of fatty hilum	76.7 (33/43)	18.9 (3/16)	61.0 (36/59)	<0.001
Hypo-or heterogeneous echogenicity	95.3 (41/43)	43.6 (7/16)	81.4 (48/59)	<0.001
Round shape	93.0 (40/43)	56.3 (9/16)	83.1 (49/59)	0.008

No., number of cases.

**Table 3:** Number of suspicious ultrasonography findings in the differentiation of cervical lymph nodes.

No. of Suspicious US Finding	% (No. with Positive Findings/Total No.)		
	Malignant	Benign	Total
0	2.3 (1/43)	37.5 (6/16)	11.9 (7/59)
1	0 (0/43)	25.0 (4/16)	6.8 (4/59)
2	14.0 (6/43)	12.5 (2/16)	13.6 (8/59)
3	30.2 (13/43)	18.6 (3/16)	27.1 (16/59)
4	53.5 (23/43)	6.3 (1/16)	40.7 (24/59)

No., number of cases; US, ultrasonography.

**Table 4:** Diagnostic performance of PET-CT, US, and combined diagnosis of PET-CT with US in the assessment of lymph node metastasis.

Type of Imaging	Imaging Diagnosis	Pathologic Diagnosis		Total (No.)	Sen. (%)	Spe. (%)	PPV (%)	NPV (%)	Accuracy (%)
		Metastasis (No.)	Benign (No.)						
PET-CT (SUVmax≥ 5.8)	Positive (No.)	35	5	40	81.4	68.8	87.5	57.9	78.0
	Negative (No.)	8	11	19					
	Total (No.)	43	16						
PET-CT + subsequent US	Positive (No.)	35	2	37	81.4	87.5	94.6	63.6	83.1
	Negative (No.)	8	14	22					
	Total (No.)	43	16						

No., number of case; NPV, negative predictive value; PPV, positive predictive value; Sen., sensitivity; Spe., specificity; US, ultrasonography.
